# Rituximab Induction and Maintenance in ANCA-Associated Vasculitis: State of the Art and Future Perspectives

**DOI:** 10.3390/jcm10173773

**Published:** 2021-08-24

**Authors:** Elena Treppo, Marco Binutti, Roberto Agarinis, Salvatore De Vita, Luca Quartuccio

**Affiliations:** Department of Medicine, Rheumatology Clinic, University of Udine, ASUFC, 33100 Udine, Italy; treppo.elena@gmail.com (E.T.); m.binutti@stdelta.com (M.B.); agarinis.roberto@gmail.com (R.A.); salvatore.devita@uniud.it (S.D.V.)

**Keywords:** vasculitis, ANCA, rituximab, B cell

## Abstract

Antineutrophil cytoplasmatic antibody (ANCA)-associated vasculitis (AAV) is a group of rare autoimmune diseases characterized by inflammation of the vascular wall. The pathogenesis of AAV is strongly associated with B cell-derived ANCAs; thus, Rituximab (RTX) has become a promising drug in the induction and maintenance treatment of AAV. The purpose of this review is to describe the efficacy and safety of RTX in the induction of remission and maintenance therapy of AAV. Herein, we summarize the randomized controlled trials that have contributed to the refinement of the use of RTX in AAV in the past decades. RTX has been proven to be effective both in new-onset disease and in relapsing disease. Although the optimal duration of AAV maintenance therapy remains unknown, the ANCAs and the B-cell repopulation may offer support for the administration of further RTX cycles (or not). The safety of RTX is comparable with cyclophosphamide, with the advantage of a low risk of malignancy and no concern for fertility. In conclusion, RTX now plays an important role in the induction and maintenance therapy of AAV. Optimizing RTX-based treatment strategies in AAV is one of the main goals of the current research in AAV.

## 1. Clinical Features and Relevance of ANCA in AAV

Antineutrophil cytoplasmatic antibody (ANCA)-associated vasculitis (AAV) is a small-sized blood vessel vasculitis. AAV encompasses a heterogeneous group of rare autoimmune diseases represented by granulomatosis with polyangiitis (GPA), microscopic polyangiitis (MPA), and eosinophilic granulomatosis with polyangiitis (EGPA) [1,2]. The terminology is linked to the presence of circulating autoantibodies, namely ANCAs, which are directed against the antigens found in the granules of neutrophils, most commonly either proteinase 3 (PR3) or myeloperoxidase (MPO) [3]. Typically, PR3-ANCA is detected in GPA (80–90% of patients), and MPO-ANCA is detected in MPA (60–85% of patients) [3]. In EGPA, the presence of ANCA shows more variability (30–60% of patients) and mainly involves MPO-ANCA [3]. AAV is a rare disease and in recent decades, several studies on its incidence and prevalence have been conducted, reporting a progressive worldwide increase [4]. Globally, the annual incidence ranges from 1.2 to 3.3 cases per 100,000 individuals, and the prevalence of AAV ranges from 4.6 to 42.1 cases per 100,000 individuals [4]. There is no clear gender predominance, though a slight male predominance among MPA compared to GPA has been reported [5,6]. Significant geographic differences have been reported among AAV subgroups, emphasising how the incidence of GPA and EGPA increases with latitude [7]. MPO-AAV and MPA are more common in Japan. PR3-AAV and GPA are more common in Europe [4]. ANCA specificity has a growing interest in the scientific community; in fact, it may fit better than clinical diagnosis for defining homogeneous groups of patients as well as for relapsing disease and clinical outcome [2].

Although AAV is a rare disease and its prevalence is geographically heterogeneous, recent studies concerning the healthcare burden of AAV reveal a high level of economic source consumption for the healthcare system [8,9]. Considering the wide spectrum of AAV organ manifestations, it is not surprising that the major cost component is the high rate of hospitalization. AAV can lead to a wide range of clinical manifestations; Table 1 shows possible multi-organ involvement. Involvement ranges from mild, such as ear, nose, and throat (ENT), to potentially life-threatening, such as alveolar haemorrhage [10].

Renal involvement is very common in GPA and MPA, especially at the onset of the disease [2]. Rapidly progressive glomerulonephritis with renal failure associated with proteinuria, microscopic haematuria, and hypertension can be the typical renal presentation [2]. Kidney biopsy typically reveals a pauci-immune focal necrotizing crescent glomerulonephritis [2]. Other histopathological features may include glomerular crescent or tubular intraepithelial infiltrates (acute inflammation) as well as glomerulosclerosis or interstitial fibrosis or tubular atrophy (chronic inflammation).

Validated scales to evaluate activity (Birmingham Vasculitis Activity Score (BVAS)) [11], damage (Vasculitis Damage Index (VDI) [12], and disease prognosis (Five-Factor Score (FFS)) [13] as well as a questionnaire about quality of life (AAV patient-reported outcomes (AAV-PRO)) [14], are extremely useful to support physicians in their choice of treatment [15].

The prognosis of AAV has greatly improved, and the 5-year survival rate has risen to around 70–80% over the past 40–50 years [16]. Several clinical factors influence the outcome, and the FFS can be applied to predict prognosis. Certainly, age and life-threatening disease at onset, for instance pulmonary-renal syndrome, influence the outcome [17]. The risk of end-stage renal disease (ESRD) is closely related with renal function at onset [18], and the findings of kidney biopsy correlate with the severity of renal involvement. The main causes of death in AAV patients are active disease and infections [19]. Additionally, among patients admitted to the intensive care unit for acute manifestations, the main causes of death are flares and infections [20,21]. Knowledge of ANCA specificity improves the classification of patients into distinct outcome categories. Many studies have focused on the sub-classification of AAV phenotypes based on different clusters (i.e., PR3-AAV, MPO-AAV, ANCA-negative). For instance, MPO-ANCA occurs in more than 80% of patients with isolated crescentic glomerulonephritis, whereas PR3-ANCA is present in more than 80% of patients with lung cavities or destructive ENT involvement [22]. Evidence of a higher risk of relapse has been found in PR3-AAV patients with renal involvement compared to non-PR3-AAV patients with renal involvement [23]. Patients with MPO-GPA show more frequently limited diseases with no severe organ involvement, a higher prevalence of subglottic stenosis, and fewer relapses than patients with PR3-GPA [22]. The highest mortality risk is observed in AAV patients with gastrointestinal (GI) and cardiovascular involvement [24]. ANCA-negative EGPA is more prone to cardiovascular involvement and experiencing higher mortality than ANCA-positive EGPA [24]. Nevertheless, up to 30% of AAV patients are ANCA-negative [24]. Several possible explanations are proposed, including the timing of ANCA testing, the presence of other pathogenic autoantibodies, the variable sensitivity of ANCA detection methods, and the presence of ANCA inhibitors that interfere with their identification.

Table 1 summarizes the main clinical features of AAV [3,25,26,27,28].

## 2. Pathogenesis of AAV: Which Are the Actors and What Is the Role of B Cells?

Regarding the pathogenesis of AAV, it can be observed that ANCA, B and T lymphocytes, endothelial cells, monocytes, and the alternative complement pathway play several roles.

It is suggested that triggering events produce an inflammatory response with an over-activation of the immune system, resulting in tissue damage [29]. Infections, genetic background, and environmental agents are involved [7].

The connection between infections and AAV is proposed by several studies. Higher relapse rates of GPA are observed in nasal carriers of *Staphylococcus aureus* [30]. Recently, Rhee et al. found associations between *Corynebacterium tuberculostearicum* and Staphylococcus species, including *S. aureus* in GPA, and demonstrated a local competitive growth between Corynebacteria and Staphylococci at the nasal mucosal level, possibly leading to GPA relapse in hosts predisposed to autoimmunity and ANCA production [31].

Ethnic studies and genome-wide association studies (GWAS) clearly support the view of a vital genetic role in the aetiology of AAV [32]. Interestingly, the associations with HLA (DQ in MPA), SERPINA1 (in GPA), and PRTN3 (in GPA) were primarily aligned with ANCA specificity rather than with the clinically defined GPA and MPA syndromes [32].

An interesting link has been established between the disease and latitudinal gradient, which may suggest that ultraviolet radiation has a role in the pathogenesis of EGPA and GPA [33]. Other potential risk factors for the development of AAV are silica exposure [34] and some drugs, including propylthiouracil, hydralazine, minocycline, and levamisole-adulterated cocaine [35]. Recently, three other new drugs (i.e., mirabegron, sofosbuvir, and nintedanib) have been identified as potential causes of AAV [36].

Following the exposure to such risk factors or other unknown events, the inflammatory response starts. The presence of a peptide that is complementary to an autoantigen (PR3 or MPO) is the trigger for the production of the anti-idiotype response (ANCAs antibodies) by the B cells [29]. This process is enhanced by imbalances in different T cell subtypes, and the cytokine–chemokine network participates in the break of tolerance and triggers autoimmunity.

Inflammatory cytokines and complement systems (i.e., the alternative complement pathway, thought C5a) primed neutrophils with the movement of MPO and PR3 to the cell surface. Then, circulating ANCAs activate neutrophils, thus conditioning an excessive and sustained presentation of PR3 or MPO at the level of the cell surface and the extracellular space. Consequently, neutrophils undergo margination, adhesion, transmigration, and oxidative stress, leading to chromatin networks in the vascular wall known as neutrophil extracellular traps (NETs) [29]. Lymphocyte function-associated antigen-1 (LFA-1) and intercellular adhesion molecule-1 (ICAM1), which are implicated in the neutrophil adhesion and migration process, might be potential therapeutic targets since the expression of LFA-1 in the neutrophils from patients with AAV is increased, and LFA-1 levels show clinical correlations [37].

Finally, NETs induce endothelial damage, apoptosis, and necrosis; moreover, the maintenance of high inflammatory levels allows for the formation of micro-abscesses and necrotizing granulomas that are rich in monocytes and macrophages [29].

Thus, B cells with their consequent ANCA production are among the major players in AAV pathogenesis, supporting the use of therapeutic strategies directly targeting B cells. Additionally, in AAV, B-cells can pathogenetically act as antigen-presenting cells as well as proinflammatory producing cells and infiltrating inflammatory cells in the tissues. Rituximab (RTX) is a chimeric monoclonal antibody that can reduce inflammation and tissue damage due to selective B cell depletion, targeting CD20 molecules on the surface of pre-B and mature B-lymphocytes. Several observational studies have provided evidence for the safety and efficacy of RTX in many autoimmune systemic diseases, including systemic vasculitis other than AAV [38,39]. In the last ten years, RTX has been successfully trailed in AAV for the induction and maintenance of remission [40,41,42,43,44,45,46,47].

## 3. Rituximab in Inducing Remission

In 2011, RTX was approved by the Food and Drug Administration (FDA) at the dose of 375 mg/m^2^ intravenous (IV) per week for 4 weeks along with glucocorticoids (GC) in the treatment of patients with GPA and MPA [45,46]. There were two randomized trials, RAVE [40] and RITUXVAS [41], that have demonstrated that RTX is not inferior to cyclophosphamide (CYC) in inducing remission in both new and relapsed patients with GPA and MPA.

The RAVE [40] study was a multicentre, randomized (1:1), double-blind trial. It compared RTX at the dose of 375 mg/m^2^ weekly for 4 weeks and oral CYC (2 mg per kilogram of body weight per day) in inducing remission in patients with GPA and MPA. The primary endpoint was a BVAS of 0 with the successful completion of prednisone taper at 6 months. Of the patients in the RTX group, 63/99 (64%) reached the primary endpoint, as compared to 52/98 (53%) patients in the control group. The difference between the two groups was not significant (*p* = 0.09) but met the criterion for non-inferiority (*p* < 0.001). The RAVE trial demonstrated that RTX was superior to CYC in relapsing disease, in fact, 34/51 (67%) patients in the RTX group reached the primary endpoint versus 21/50 (42%) in the control group, *p* = 0.01. In addition, a difference in the loss of ANCA reactivity was observed: 51% of patients in the RTX group became PR3-ANCA negative versus 17% of the control group (*p* < 0.001). There were no significant differences in the number of total adverse events between the two groups; nevertheless, more patients in the control group had one or more adverse events (33% versus 22%, *p* = 0.01). At 18 months, it was confirmed that RTX is not inferior to CYC (*p* < 0.001) in achieving remission [48].

The RITUXVAS [41] study was a 12-month, randomized (3:1) controlled trial that involved 44 patients with newly diagnosed renal involvement of AAV. Successively, it was extended to a 24-month randomized controlled trial [49]. Patients received GC plus either RTX (375 mg/m^2^ per week for 4 weeks) with two CYC IV pulses or CYC IV for 3–6 months followed by AZA. There was no difference in the primary composite outcome of death, end-stage renal disease (ESRD), and relapse between the two groups at 24 months. Cumulative renal survival rates reached 93% in the RTX group and 100% in the control group (*p* = 0.39). Adverse event rates were comparable in the two groups. The abovementioned randomized controlled trials investigating rituximab as induction in AAV are summarized in Table 2.

Interestingly, even if the primary objective of PEXIVAS trial was to verify the advantage of plasma exchange (PEX) combined with RTX or CYC in AAV [50], it was the first trial in which patients with GPA or MPA suffering from severe renal vasculitis or diffuse alveolar haemorrhage received RTX associated with PEX. Importantly, the reduced-dose regimen of glucocorticoids tested in PEXIVAS was noninferior to a standard-dose regimen with respect to death or ESRD, while serious infections at 1 year were less common in the reduced-dose group than in the standard-dose group [48]. Thus, a glucocorticoid regimen is the most important modifiable variable that conditions the risk of infection, regardless the drug used in the induction.

Of note, published trials on RTX have not included EGPA. EGPA remains a relatively understudied group of AAV, owing to its relative rarity and phenotypic difference from GPA and MPA. Recently, two systematic reviews about the use of RTX in EGPA have been published [51,52], according to which RTX shows efficacy in inducing remission in both new-onset and relapsing EGPA. Results showed a greater benefit in EGPA with ANCA positivity. Nevertheless, more studies focusing on the use of RTX in EGPA are needed.

## 4. Rituximab in Maintaining Remission

RTX is also recommended in maintaining remission in patients with GPA and MPA [2,53].

The MAINRITSAN trial [42] randomized 115 patients with newly diagnosed (92/115) or relapsing (23/115) AAV (excluding EGPA) who received a maintenance regimen based on either RTX (500 mg on days 0 and 14 and at months 6, 12, and 18) or a daily azathioprine (AZA) dose until month 22. All of the patients were in complete remission after a CYC-GC regimen, and the primary endpoint was the rate of major relapse at month 28. At month 28, major relapse had occurred in 17 patients in the AZA group (17/58, 29%) and in 3 patients in the RTX group (3/57, 5%) (HR for relapse, 6.61; 95% CI, 1.56 to 27.96; *p* = 0.002). The study showed the superiority of this RTX regimen over azathioprine (AZA) in relapse prevention up to a follow-up of 60 months [54].

The MAINRITSAN2 trial [43] compared the fixed-schedule RTX (500 mg on days 0 and 14 and at months 6, 12, and 18) with an individually tailored RTX maintenance regimen (500 mg on days 0 and 14, further 500 mg based on a 3-monthly measure of ANCA and B cells but only in cases where CD19 + B lymphocytes or ANCA reappeared or ANCA titre rose markedly based on trimestral testing until month 18). The low relapse rates observed in the two arms after 28 months did not differ significantly (13/81 relapsed patients in tailored-infusion versus 8/81 relapsed patients in fixed-schedule-infusion, *p* = 0.22) and were comparable with the MAINRITSAN trial. The MAINRITSAN2 trial demonstrated that it is possible to maintain remission with fewer infusions. Nevertheless, the role of ANCA as a marker of relapse remains a source of debate, and relapses have been also observed in cases of ANCA negativity and B cell depletion; therefore, actually fixed interval dosing has been recommended [53]. In selected patients after 2 years of maintenance therapy, relapse risk remains high, or relapse may be very risky due to the type of clinical involvement, and extended RTX maintenance therapy should be considered (500–1000 mg every 6–12 months for up to 5 years) [53]. 

The recently published MAINRITSAN3 trial [44] shows that prolonging RTX treatment (500 mg infused every 6 months for an additional 18 months) after an initial 18-month maintenance regimen was effective in sustaining remission. The relapse-free survival at 28 months was 96% in the RTX group versus 74% in the control group (HR 7.5, CI, 1.67 to 33.7, *p* = 0.008). The relapse occurred in 2/50 patients in the RTX group versus 12/47 patients in the control group. Among the 12 relapsed patients in the placebo group, 10 (83%) had GPA, 2 (17%) had MPA, 6 were having their first relapse, and all had ANCA positivity (10 PR3-ANCA, 2 MPO-ANCA positivity). In MAINRITSAN3, the relapses seemed to occur more frequently in patients with PR3-AAV than in patients with MPO-AAV. This could suggest again the benefits of long-term RTX administration in this subpopulation [44]. Overall, it is becoming increasingly clear that AAV patients with a relapsing disease need different strategies compared to those with a non-relapsing disease.

The RITAZAREM trial [46,55] is an international, multicentre, open-label, randomized controlled trial recruiting only the subgroup of patients with relapsing AAV. It aims to demonstrate the superiority of RTX over AZA in the prevention of relapses in AAV with relapsing disease. In the RITAZAREM trial, relapsing AAV patients were recruited and received induction therapy with RTX and GCs. If patients achieved remission by month 4, they were randomized in a 1:1 ratio and received a maintenance therapy with either RTX (1000 mg every 4 months for 5 doses) or AZA (2 mg/kg/day). Results show that RTX is superior to AZA in preventing disease relapse with a HR of 0.36 (95% CI 0.23–0.57, *p* < 0.001). By the 24th month after randomization, relapse occurred in 11/85 (13%) of patients in the RTX group compared to 32/85 (38%) in the AZA group. At least one severe adverse event (SAE) occurred in 19/85 (22%) patients in the RTX group and in 31/85 (36%) patients in the AZA group. Hypogammaglobulinemia (IgG < 5 g/L) and non-severe infections were reported in 25/85 (29%) and 42/85 (49%) patients in the RTX group, respectively, compared to 21/85 (25%) and 41/85 (48%) in the AZA group. Notably, long-term data revealed that the effect of higher-dose RTX is not sustained over time, and relapses are very common in AAV, regardless of maintenance agent used [55].

The above-mentioned trials are in Table 3.

## 5. Towards a Patient-Tailored Use of RTX in AAV

The optimal long-term strategy after fixed-schedule RTX has not been clarified. Several studies have investigated the biomarkers of disease relapse of AAV after RTX treatment. Considering the experience with RTX therapy in other autoimmune diseases, the residual B cells have been investigated. Circulating B cells are detectable in all timepoints using high sensitivity flow cytometry instead of standard flow cytometry [56], and this was linked to the tendency of AAV to relapse, at least in a fraction of patients. Furthermore, specific B-cell populations have different roles in AAV. A reduced risk of relapse has been demonstrated in case of naïve B cell population at 6 months after RTX [57]. By contrast, an increased risk of relapse has been associated with the presence of circulating CD27+ CD38+ plasma cells during disease remission [58]. A combination of B-cell targeting therapies, such as RTX and belimumab, might improve the remission maintenance in PR3-ANCA positive patients [NCT03967925]. Other risk factors for relapsing disease are GPA subtypes, ANCA positivity, especially PR3-ANCA, upper respiratory involvement, and previous relapses [59]. Up to 30% of patients can show a relapsing or even refractory disease [60], which first requires transition from CYC to RTX or from RTX to CYC. Strategies employed in this group of diseases include a combination of RTX and CYC [61]; RTX, CYC, and PEX [62]; the use of PEX or high-dose intravenous immunoglobulins [63]; or, more recently, complement inhibition [64].

## 6. Safety of Rituximab in AAV

Along with a better prognosis for AAV patients, the safety of long-term therapies has progressively become a primary focus of interest. The optimal balance between AAV therapy risks and benefits is a well-known and persistent challenge.

Infections and infusion reactions are the most common adverse events. The issue of the infections remains open since they still represent the most important cause of hospitalization and mortality, which is sometimes related to secondary hypogammaglobulinemia [65].

Hypogammaglobulinemia following RTX is not uncommon and more likely in patients with high GCs and CYC exposure and low IgG levels at baseline [66,67]. Hypogammaglobulinemia is typically defined as a serum IgG level below 600 mg/dL and can be further stratified as mild (400–599 mg/dL), moderate (200–399 mg/dL), and severe (0–199 mg/dL). Recommendations for the management of secondary hypogammaglobulinaemia due to B cell targeted therapies in autoimmune rheumatic diseases has recently been published although the strength of the recommendations was limited by the low quality of the evidence and the absence of randomized controlled trials [67]. Notably, immunoglobulin replacement can reduce the infection rate, but not the severe infection rate, in patients with a recurrence of infections [66]. Prophylaxis for *Pneumocystis jirovecii* during RTX therapy should be considered in all patients, both in the induction and maintenance regimen, and flu and pneumonia vaccination should be encouraged. In fact, the rate of infections can be lowered by trimethoprim–sulfamethoxazole in AAV undergoing RTX [68,69]. Additionally, RTX is considered a risk factor for poor outcomes in the case of SARS-CoV-2 infection [70,71,72]; thus, AAV patients who need RTX therapy should be recommended to undergo SARS-CoV-2 vaccination before RTX, if possible [70,71].

The incidence of infection with RTX is largely conditioned by the use of concomitant corticosteroids, previous treatments, and comorbidities [44]. The incidence of infectious complications with RTX was as high as with CYC, and this issue needs further investigation [40]. The concomitant use of corticosteroids together with lung comorbidity and diabetes are probably the main drivers for the risk of infections in AAV under RTX, and novel treatment strategies should be aimed to address the issue of sparing corticosteroids in the short and long term [73]. Several ongoing randomized controlled trials are aiming to optimize RTX dosage, possibly in combination with other drugs (CYC or belimumab or avacopan), and to minimize or possibly avoid glucocorticoids (Clinicaltrials.gov. NCT03942887; NCT03967925; NCT03920722; NCT032290456; NCT02749292; NCT02994927). In this regard, very recently, a Japanese phase 4, multicentre, open-label, randomized, noninferiority trial compared two corticosteroid regimens (reduced-dose prednisolone 0.5 mg/kg/day versus high-dose prednisolone 1 mg/kg/day) plus RTX 375 mg/m^2^/week, four doses in 140 patients with newly diagnosed AAV without severe glomerulonephritis or alveolar haemorrhage. In this trial, there was no difference in the primary endpoint, which was the remission rate at 6 months. Importantly, serious adverse events and, in particular, serious infections occurred at a significantly lower rate in the reduced-dose prednisolone arm [74]. Overall, positive results, if confirmed, will be of major value to improving the safety of the induction regimen with RTX.

Overall, the rate of infusion reactions was low (5%) [40,75] Infusion related-reactions (IRR) are usually mild to moderate, though fatal evolutions have been reported [40,75]. The most common IRR are fever, rash, itching, and headache [40,75]. More severe IRR includes angioedema, hypotension, and bronchospasm [40,75].

Late delayed neutropenia can usually be observed 6–8 months after RTX treatment, and it is more frequent in GPA (23%) than in lupus or rheumatoid arthritis [76]. Late-onset neutropenia can be observed in patients with a RTX maintenance regimen and usually recovers without treatment [53]. It is rarely associated with serious infections, which is different from early neutropenia, which is less frequent, but is possibly complicated by serious infections [76].

Data from the European Vasculitis Study Group (EUVAS) demonstrated a 2.8-fold incidence of an increased risk for non-melanoma skin cancer (NMSC) in AAV patients and a non-significant standardized incidence ratio (SIR) for non-NMSC (1.30) than general population expectations [77]. Recently, a propensity score-matched analysis of a nationwide study demonstrated that age, male sex, GPA sub-type, and CYC therapy was associated with cancer risk in AAV [78]. The malignancy risk in patients with AAV was lower in RTX-treated patients than in CYC-treated patients [79]. Notably, RTX treatment was not associated with an increased malignancy risk compared to the general population [79].

Despite the lack of head-to-head trials, retrospective studies supported that similar biologic RTX was as effective and safe as an originator in induction and remission maintenance in patients with AAV [80,81]. A recent systematic review comparing the four-dose (375 mg/m^2^ intravenously weekly) versus the two-dose (1000 mg intravenously biweekly) regimens in AAV did not find any differences for either efficacy or safety [82].

## 7. Conclusions

**Figure 1:**
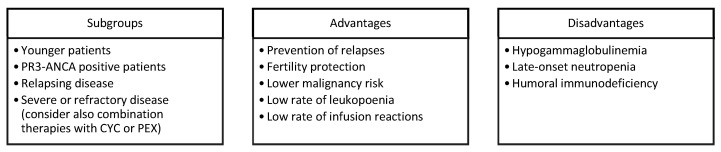
Major drivers, PROS, and CONS for choosing RTX in AVV. Legend: PR3, proteinase 3; CYC, cyclophosphamide; PEX, plasma exchange.

RTX plays an important role in AAV induction and maintenance therapy, especially in some subgroups of patients (Figure 1). After the introduction of CYC, which significantly improved the survival of AAV [83], the efficacy of RTX in AAV successfully addressed the issues of fertility preservation and the increased risk of malignancy under CYC. A maintenance therapy with RTX can decrease the rate of relapse and, as a consequence, the cumulative dose of corticosteroids. The optimal duration of RTX maintenance remains unknown, and further studies are required. The ANCA antibody seems to be a promising biomarker to guide RTX maintenance since an increased ANCA titre could reflect the incomplete B cell depletion and subclinical disease activity that may still require B-cell depletion [54]. RTX can be considered a long-term treatment for AAV with correctable side effects. Optimizing B cell-depleting therapy and steroid-sparing regimens is the next step towards further improvements in both the mortality rate and quality of life of AAV patients.

## Figures and Tables

**Table 1:** Clinical and laboratory characteristics of patients with antineutrophil cytoplasmatic antibody (ANCA)-associated vasculitis.

Clinical Manifestations	MPA	GPA	EGPA
Constitutional symptoms	Fever, Weight Loss, Fatigue, Arthralgia, Myalgia		
	55–80%	70–100%	30–50%
Skin	Palpable Purpura, Nodules, Pseudourticarial Rash, Livedo Reticularis, Ulcers		
	35–60%	10–50%	50–70%
ENT	Infrequent	Frequent (60–80%): Destructive Sinusitis, Saddle-Nose Deformity, Crusting Rhinitis, Nasal Septum Deformity, Otitis Media	Allergic Rhinitis, Sinus Polyposis
Lung	Frequent (60–80%): Cough, Haemoptysis, Dyspnoea, Interstitial Lung Pattern, Alveolar Haemorrhage	Frequent (60–80%): Non-Migratory Nodule or Infiltrates, Excavated Nodules, Bronchial And/or Subglottic Stenosis	Asthma (Approximately 100%), Migratory Nodules or Infiltrates, Eosinophil Pleural Effusion
Kidney	Proteinuria, Haematuria, Renal Failure		
	Frequent (80%): Glomerulonephritis	Frequent (60–80%): Glomerulonephritis	Possible (20%)
Neurologic	Mononeuritis Multiplex, Polyneuropathy, Cranial Nerves Disorders, Pachymeningitis		
	Possible (35%)	Possible (25%)	Frequent (65–75%)
Heart	Myocarditis, Pericarditis, Ischemia		
			Possible (10–50%): From Asymptomatic to Cardiomyopathy
Eye	Uveitis, Conjunctivitis, Episcleritis		
		Mono or Bilateral Proptosis, Orbital Tumour	
Venous thrombosis	7–8%		
Laboratory	Increase ESR and CRP, Anaemia, Thrombocytosis		
			Eosinophilia
cANCA/PR3	10–20%	80–90%	
pANCA/MPO	60–85%	0–10%	30–60%, usually pANCA/MPO

Legend: MPA, microscopic polyangiitis; GPA, granulomatosis with polyangiitis; EGPA, eosinophilic granulomatosis with polyangiitis; ENT, ear, nose and throat; ESR, erythrocyte sedimentation rate; CRP, C-reactive protein; ANCA, antineutrophil cytoplasmatic antibody; cANCA, cytoplasmic ANCA pattern; PR3, proteinase 3; pANCA, perinuclear ANCA pattern; MPO, myeloperoxidase.

**Table 2:** Main characteristics and results of randomized trials using rituximab as induction in AAV.

Name	Population	Number of Patients	Primary Endpoint	Results	Other Findings
RAVE [40]	N = 197 pts; GPA or MPA; new onset (49%) or relapsing disease; ANCA+	RTX arm: 99 pts received 4 weekly RTX 375 mg/m^2^; Control arm: 98 pts received PO CYC followed by AZA; same GCs regimen; randomized 1:1	Remission of disease without GCs at 6 months	(1) RTX was noninferior to CYC (64% vs. 53%) at remission induction at 6 months (*p* < 0.001) (2) RTX was superior to CYC (67% vs. 42%) in relapsing disease (*p* = 0.01)	(a) 50% of pts in RTX arm became negative for PR3–ANCA, as compared with only 17% in the control arm; (b) similar AEs
RITUXVAS [41]	N = 44 pts; GPA or MPA; new onset of renal AAV	RTX arm: 33 pts received two doses CYC IV plus 4 weekly RTX 375 mg/m^2^; Control arm: 11 pts received IV CYC followed by AZA; same GCs regimen; randomized 3:1	Sustained remission rates at 12 months and severe AEs	(1) Equivalent results in achieving sustained remission (76% vs. 82%, *p* = 0.68) 2)Severe adverse events were similar (42% vs. 36%, *p* = 0.77)	(a) Sustained remission rates were high in both groups

Legend: MPA, microscopic polyangiitis; GPA, granulomatosis with polyangiitis; RTX, rituximab; AZA, azathioprine; CYC, cyclophosphamide; AAV, ANCA-associated vasculitis; GCs, glucocorticoids; AEs, adverse effects.

**Table 3:** Main characteristics and results of randomized trials using rituximab as maintenance in AAV.

Name	Population	Number of Patients	Primary Endpoint	Results	Other Findings
MAINRITSAN [42]	N = 115 pts; GPA or MPA in remission of disease after CYC; ANCA+	RTX arm: 57 pts received RTX (500 mg every 6 months); Control arm: 58 pts received AZA; randomized 1:1	Rate of major relapse at month 28	(1) Lower relapse rate in RTX arm (5% vs. 29%, HR for relapse 6.61, IC 95%: 1.56–27.96, *p* = 0.002)	(a) Similar AEs (*p* = 0.92)
MAINRITSAN2 [43]	N = 162 pts; GPA or MPA in remission of disease; ANCA+ or ANCA-	Tailored-arm: 81 pts patients received a 500 mg RTX infusion at randomisation, then in case of change in ANCA status or CD19+ B cell counts exceeded 0/mm^3^; Control arm: 81 pts receiveda fixed 500 mg RTX infusion on days 0 and 14 postrandomisation, then 6, 12, and 18 months after the first infusion; randomized 1:1	Number of relapses or worsening disease (BVAS > 0) at month 28	(1) Equivalent results in number of relapses [21 pts had suffered 22 relapses: 14/81 (17.3%) in 13 tailored-infusion recipients and 8/81 (9.9%) in 8 fixed-schedule patients (*p* = 0.22)]	(a) AAV relapse rates did not differ significantly; (b) individually tailored-arm patients received fewer rituximab infusions
MAINRITSAN3 [44]	N= 97 pts; GPA or MPA in sustained remission after RTX-maintenance therapy; pts must have successfully completed the MAINRITSAN2 trial without any major relapses	RTX arm: 50 pts received additional 2 years of RTX over 18 months (4 infusions); Control arm: 47 pts received placebo; randomized 1:1	Relapse-free survival at month 28	(1) Relapse-free survival was higher in RTX arm at month 28 (96% vs. 74%, HR 7.5, CI: 1.67–33.7, *p* = 0.008)	(a) Major relapse-free survival estimates at month 28 were 100% in RTX arm versus 87% in control arm (*p* = 0.009); (b) lower relapse rate in RTX arm (4% versus 26%); (c) no increase in AEs in extended RTX (24% versus 30%) (d) in the placebo arm, relapse is much more common in PR3-ANCA positive pts
RITAZAREM [46,55]	N = 190 pts; relapsed GPA or MPA re-induced with RTX (4 weekly RTX 375 mg/m^2^) and in remission of disease at month 4 (N = 170)	RTX arm: 85 pts received RTX (1000 mg every 4 months for 5 doses); Control arm: 85 pts received AZA; randomized 1:1	Time to disease relapse reported at 24 months	(1) RTX was superior to AZA in relapsing disease with preliminary overall HR estimate of 0.36 (CI 95%: 0.23–0.57, *p* < 0.001)	(a) No increase AEs in RTX arm (22% versus 36%); (b) relapse is very common in both arms; (c) the effect of higher-dose RTX is not sustained in long term

Legend: MPA, microscopic polyangiitis; GPA, granulomatosis with polyangiitis; RTX, rituximab; AZA, azathioprine; CYC, cyclophosphamide; AAV, ANCA-associated vasculitis; AEs, adverse effects; HR, hazard ratio.

## Data Availability

No new data were created or analyzed in this study. Data sharing is not applicable to this article.
